# Preparation of Thermo-Responsive and Cross-Linked Fluorinated Nanoparticles via RAFT-Mediated Aqueous Polymerization in Nanoreactors

**DOI:** 10.3390/molecules22020152

**Published:** 2017-01-25

**Authors:** Jiachen Ma, Luqing Zhang, Bing Geng, Umair Azhar, Anhou Xu, Shuxiang Zhang

**Affiliations:** 1 Shandong Provincial Key Laboratory of Fluorine Chemistry and Chemical Materials, School of Chemistry and Chemical Engineering, University of Jinan, Jinan 250022, China; majiachen9209@163.com (J.M.); ujnb636@163.com (L.Z.); chm_gengb@ujn.edu.cn (B.G.); umair.azhar9922@gmail.com (U.A.); 2 Shandong Engineering Research Center for Fluorinated Material, University of Jinan, Jinan 250022, China

**Keywords:** RAFT polymerization, nanoreactor, aqueous polymerization, cross-linking, temperature sensitivity

## Abstract

In this work, a thermo-responsive and cross-linked fluoropolymer poly(2,2,2-Trifluoroethyl) methacrylate (PTFEMA) was successfully prepared by reversible addition-fragmentation chain transfer (RAFT) mediated aqueous polymerization with a thermo-responsive diblock poly(dimethylacrylamide-*b*-*N*-isopropylacrylamide) (PDMA-*b*-PNIPAM) that performed a dual function as both a nanoreactor and macro-RAFT agent. The cross-linked polymer particles proved to be in a spherical-like structure of about 50 nm in diameter and with a relatively narrow particle size distribution. ^1^H-NMR and ^19^F-NMR spectra showed that thermo-responsive diblock P(DMA-*b*-NIPAM) and cross-linked PTFEMA particles were successfully synthesized. Influence of the amount of ammonium persulfate (APS), the molar ratio of monomers to RAFT agent, influence of the amount of cross-linker on aqueous polymerization and thermo-responsive characterization of the particles are investigated. Monomer conversion increased from 44% to 94% with increasing the molar ratio of APS and P(DMA-*b*-NIPAM) from 1:9 to1:3. As the reaction proceeded, the particle size increased from 29 to 49 nm due to the consumption of TFEMA monomer. The size of cross-linked nanoparticles sharply decreased from 50.3 to 40.5 nm over the temperature range 14–44 °C, suggesting good temperature sensitivity for these nanoparticles.

## 1. Introduction

In recent years, the study of fluoropolymers has gained tremendous attention because of their versatility. Excellent chemical resistance, thermal stability and other special properties enable them to be widely used in the fields of leather, textiles, building, and coating. In addition, many fluorinated polymers and small molecules have been widely used in medicine and drug delivery because of their biocompatibility and non-toxicity in vivo [1,2,3]. For example, sitagliptin, a selective, potent dipeptidyl peptidase IV DPP-4 inhibitor, is the active ingredient in JANUVIA^®^ and JANUMET^®^ (a fixed dose combination with the antidiabetic agent metformin), which both recently received approval for the treatment of type 2 diabetes by the FDA [4]. The most widely used method for the preparation of polymer nanoparticles is heterogeneous polymerization, including emulsion polymerization and dispersion polymerization. Aqueous polymerization is a relatively economical and versatile tool to produce nanoparticles with a low sensitivity to impurities [5]. Usually emulsifiers are used to stabilize such type of emulsions. These emulsifiers are very toxic and also need to be removed from the final product, which imparts extra costs. Based on the reasons above, emulsifier-free aqueous polymerization was developed. The main drawback of emulsifier-free aqueous polymerization was the broad particle size distribution (PSD) with little or no control over particle size.

With the development of controlled/living radical polymerization (CLRP) the problem of large dispersity (Ð) and uncontrolled molecular weight has been overcome to a large extent [6]. Researchers simultaneously found that it is a good way to produce particles with narrow size distribution. CLRP include nitroxide-mediated polymerization, atom transfer radical polymerization and reversible addition-fragmentation chain transfer (RAFT) polymerization [7,8,9]. Among these methods, RAFT polymerization has distinct advantages compared to others. It can be applied to synthesize polymers or copolymers with narrow molecular weight distribution for most monomers amenable to radical polymerization. The molecular weight of the final product can be predicted from the ratio of monomer consumed to chain transfer agent. In addition, there is compatibility with a vast range of functional monomers, solvents and initiators. Also, there is no undesired metal species introduced during the RAFT polymerization process [9,10].

Considerable attention has been directed towards the synthesis of polymer nanoparticles via RAFT polymerization in dispersed aqueous medium (e.g., emulsion, miniemulsion) due to its capability of controlled molecular weight and dispersity [6,11,12,13,14,15,16]. Firstly, homopolymers and copolymers are synthesized and then used in aqueous RAFT polymerization. During this process amphiphilic block copolymers not only provide an ideal setting for polymerization to prevent the aggregation of nanoparticles but also acts as a reactant to produce products with well-controlled dispersity [6]. Many organic reactions have been carried out in nanoreactors [17,18]. These nanoreactors can provide a nano-sized space for the reaction to take place. For P(DMA-*b*-NIPAM) copolymer, the PNIPAM block is hydrophilic and water-soluble below its lower critical solution temperature (LCST ~36 °C) [19] while above its LCST it forms self-stabilized monodisperse nanoreactors [6]. The main advantages of carrying out CLRP using nanoreactors are the following: (1) easy to form micelles in water with better emulsion stability; (2) pure products can be obtained with low dispersity and no residual emulsifier [20,21,22,23].

In this study, thermo-responsive and cross-linked poly((2,2,2-trifluoroethyl)methacrylate) nanoparticles were successfully prepared. Firstly, a thermo-responsive diblock copolymer based on RAFT solution polymerization was synthesized. The diblock copolymer containing a RAFT end group is used for RAFT-mediated aqueous polymerization to prepare cross-linked nanospheres due to its self-emulsifying property. Homopolymer PNIPAM has been extensively studied for drug delivery applications because of its lower critical solution temperature (LCST) (at ~32 °C) [24]. However, PNIPAM homopolymers suffer from syneresis and lack of biodegradability. The fluorinated core-shell structure can retain temperature-sensitive properties and water absorption along with the uniformity and stability of nanospheres. The strong hydrophobicity of the fluorinated core can enhance the performance of the loading of hydrophobic drug. To our knowledge, this is the first time to synthesize thermo-responsive fluoropolymer nanospheres. It may have an attractive application prospect in drug controlled release.

## 2. Results and Discussion

### 2.1. Synthesis and Characterization of PDMA-S-(C=S)-S-C_12_H_25_ and P(DMA-b-NIPAM)-S-(C=S)-S-C_12_H_25_

**Figure 1:**
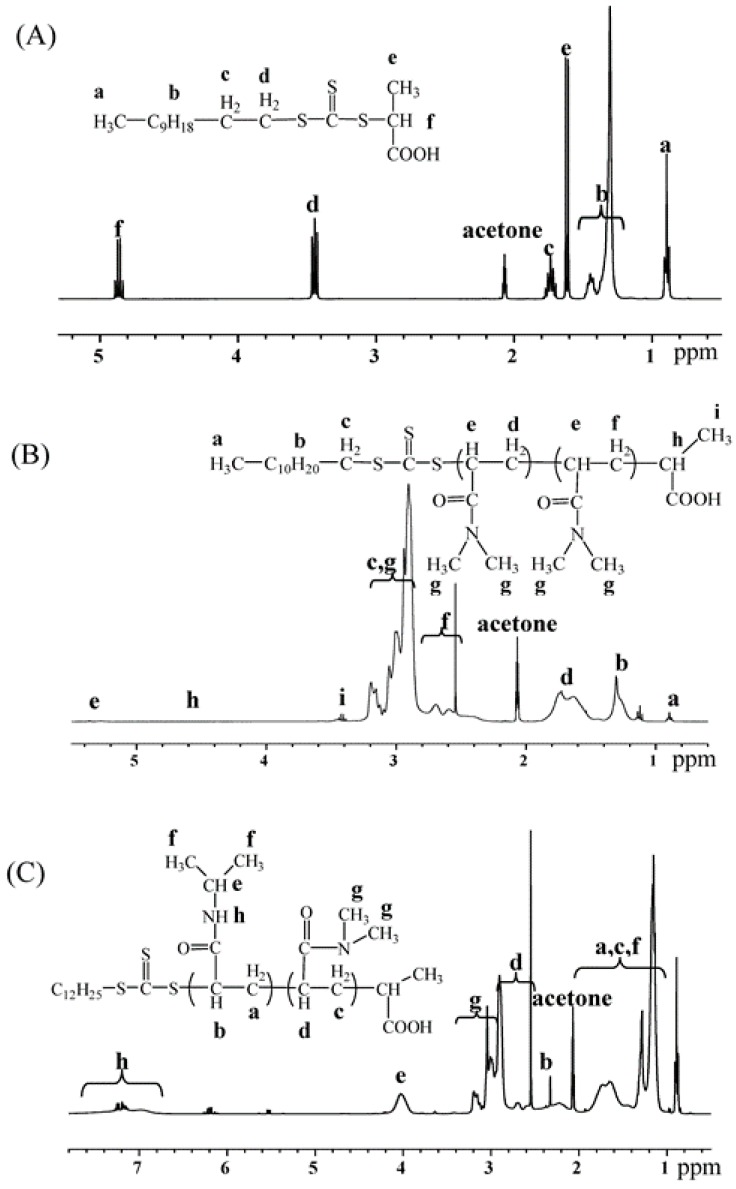
^1^H-NMR spectra of (**A**) HOOCCH(CH_3_)-S-(C=S)-S-C_12_H_25_; (**B**) macro-CTA PDMA_73_-S-(C=S)-S-C_12_H_25_; (**C**) copolymer P(DMA_73_-*b*-NIPAM_77_)-S-(C=S)-S-C_12_H_25_ in acetone.

P(DMA-*b*-NIPAM)-S-(C=S)-S-C_12_H_25_ was synthesized via a two-step RAFT solution polymerization of dimethylacrylamide (DMA) and *N*-isopropylacrylamide (NIPAM) with the trithiocarbonate RAFT agent 2-([(dodecylsulfanyl)carbonothioyl]sulfanyl)propanoic acid. As the ^1^H-NMR spectra in Figure 1 shows, all proton signals are assigned. In Figure 1B, the feature signals of the polydimethylacrylamide (PDMA) segment are seen (1.42–1.91 ppm –C*H*_2_– backbone PDMA; 2.30–2.78 ppm –C*H*– backbone PDMA; 2.78–3.27 ppm dimethyl group of NMe_2_, PDMA) [25]. For the diblock ^1^H-NMR spectrum in Figure 1C, the feature signals of PDMA still remain, while the feature signals of poly(*N*-isopropylacrylamide) (PNIPAM) now appear (3.80–4.22 ppm –C*H*– in side chain PNIPAM; 6.67–7.75 ppm –N*H*–, PNIPAM) [6,25]. The signal at 0.95 ppm is attributed to the methyl protons of the RAFT agent, which indicates that the structure of the final polymer still keeps the functional group of the RAFT agent. The chemical structure of the chain transfer agent (CTA), macro-RAFT agent and diblock are thus confirmed from the ^1^H-NMR.

**Figure 2:**
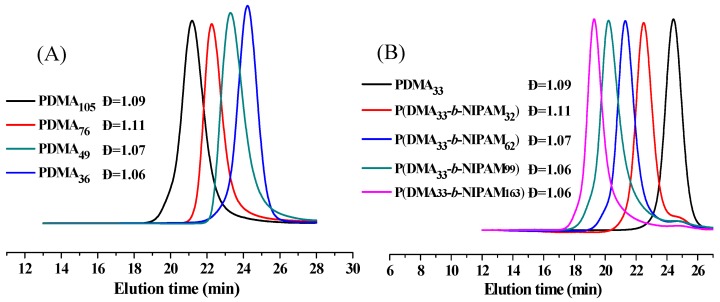
Gel permeation chromatography (GPC) traces of homopolymer or diblock copolymer eluted by THF. (**A**) macro-RAFT agent of PDMA-S-(C=S)-S-C_12_H_25_ with different degree of polymerization; (**B**) P(DMA-*b*-NIPAM)-S-(C=S)-S-C_12_H_25_ copolymers prepared with the molar ratios of NIPAM/PDMA_33_-S-(C=S)-S-C_12_H_25_ at 43:1, 80:1, 140:1 and 250:1. Polymerization conditions: PDMA_33_-S-(C=S)-S-C_12_H_25_:[AIBN] = 7:1, 65 °C, 20 h.

Table 1 and Table 2 show the data of the synthesis of poly(*N*,*N*-dimethylacrylamide) (PDMA) and PDMA-*b*-PNIPAM. Figure 2A shows the gel permeation chromatography (GPC) traces of the macro-RAFT agent of PDMA-S-(C=S)-S-C_12_H_25_ with a different ratio of monomer and RAFT agent, resulting in a different degree of polymerization. Based on the GPC traces eluted by THF, the different molecular weights ranging from 3900 to 10,800 g/mol with low dispersity (<1.1) for PDMA-S-(C=S)-S-C_12_H_25_ are obtained. Figure 2B shows the GPC traces of P(DMA-*b*-NIPAM)-S-(C=S)-S-C_12_H_25_ synthesized by the same macro-RAFT agent. Diblock copolymers ranging from 7200 to 22,000 g/mol with low dispersity were successfully obtained. These results confirm the well-controlled RAFT polymerization.

### 2.2. Crosslinking Polymerization of TFEMA in Nanoreactors Formed by P(DMA-b-NIPAM)-S-(C=S)-S-C_12_H_25_

Thermo-responsive block polymers have been extensively studied for drug delivery and extraction. Gupta’s group reported that thermo-responsive triblock polymer poly-[(propylenesulfide)-*b*-(*N*,*N*-dimethylacrylamide)-*b*-(*N*-isopropylacrylamide)] (PPS-*b*-PDMA-*b*-PNIPAAM) can form micelles at room temperature (25 °C). The micelles preloaded with the model drug Nile Red will form hydrogels upon heating to physiologic temperature (37 °C), and the resulting hydrogels demonstrated reactive oxygen species (ROS)- dependent drug release [26]. Duhamel et al. demonstrated that poly(ethylene glycol)-*b*-poly(2-(2-methoxyethoxy) ethyl methacrylate) (PEG-*b*-PMEO_2_MA) has the ability to extract oil from oil sands [27].

**Figure 3:**
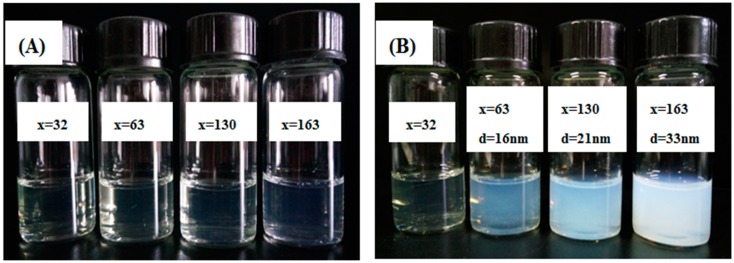
(**A**) Optical photographs of P(DMA_33_-*b*-NIPAM_x_)-S-(C=S)-S-C_12_H_25_ (x = 32, 63, 130, 163) aqueous solution with 2.0 wt % concentration at 25 °C; (**B**) Optical photographs at 40 °C and diameter measured by dynamic light scattering (DLS).

Optical photographs of the thermo-responsive block polymers that we synthesized are shown in Figure 3; the solution was transparent at 25 °C. When the temperature was raised to 40 °C, the P(DMA_33_-*b*-NIPAM_x_)-S-(C=S)-S-C_12_H_25_ (x = 63, 130, 163) solution became opaque, while P(DMA_33_-*b*-NIPAM_32_)-S-(C=S)-S-C_12_H_25_ showed no change. The thermo-responsive diblock P(DMA-*b*-NIPAM)-S-(C=S)-S-C_12_H_25_ can completely dissolve in water below the LCST of the PNIPAM block. The behavior of the PNIPAM block changes from soluble into insoluble once the temperature is above the LCST, resulting in the aggregation of PNIPAM segments to form nanoreactors, and hydrophilic segment PDMA acts as a stabilizer for the nanoreactors. The nanoreactors can encapsulate TFEMA monomers and cross-linker EGDMA for reaction. Intensity weighted distributions of the hydrodynamic diameter were obtained by dynamic light scattering (DLS). The micelle hydrodynamic diameter increased from 16 to 33 nm while the degree of polymerization of the PNIPAM segment increased from 63 to 163. The PNIPAM segments of P(DMA_33_-*b*-NIPAM_32_)-S-(C=S)-S-C_12_H_25_ are too short to stabilize the micelles.

**Figure 4:**
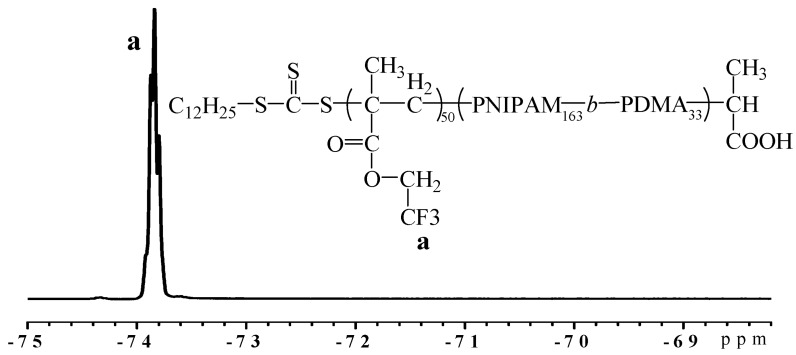
^19^F-NMR spectrum of P(DMA_33_-*b*-NIPAM_163_-*b*-TFEMA_50_)-S-(C=S)-S-C_12_H_25_ nanoparticles.

**Figure 5:**
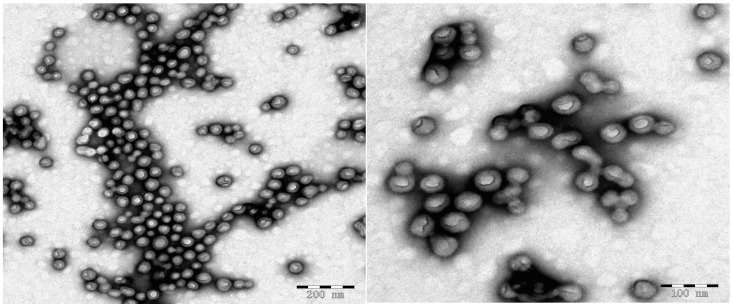
Transmission electron microscopy (TEM) images for the final latex of RAFT-mediated aqueous polymerization of TFEMA in the presence of P(DMA-*b*-NIPAM)-S-(C=S)-S-C_12_H_25_. Polymerization conditions: P(DMA_46_-*b*-NIPAM_31_)-S-(C=S)-S-C_12_H_25_:[APS] = 3:1, P(DMA_46_-*b*-NIPAM_31_)-S-(C=S)-S-C_12_H_25_:[TFEMA] = 1:200, [EGDMA]:[TFEMA] = 5:100, 70 °C, 120 min.

**Figure 6:**
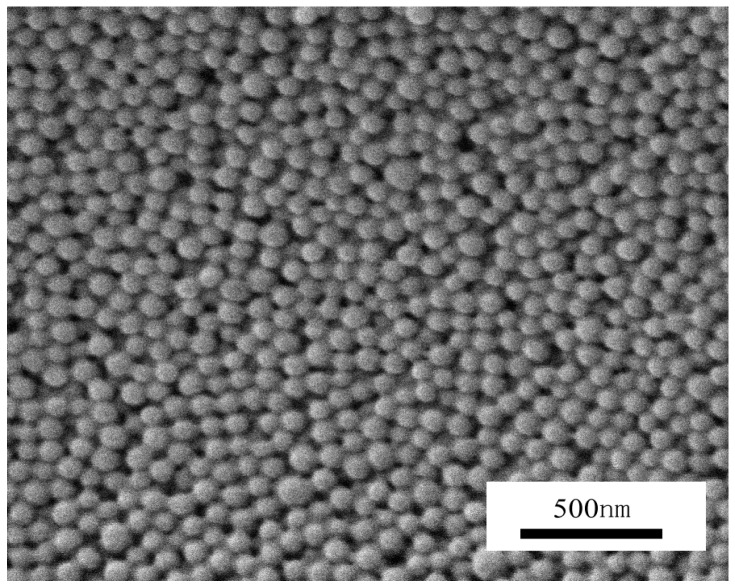
Scanning electron microscopy (SEM) image for the lyophilized triblock copolymer P(DMA_33_-*b*-NIPAM_32_-*b*-TFEMA_500_)-S-(C=S)-S-C_12_H_25_. Polymerization conditions: P(DMA_33_-*b*-NIPAM_32_)-S-(C=S)-S-C_12_H_25_:[APS] = 3:1, P(DMA_33_-*b*-NIPAM_32_)-S-(C=S)-S-C_12_H_25_:[TFEMA] = 1:500, [EGDMA]:[TFEMA] = 5:100, 70 °C, 120 min.

The aqueous solution containing P(DMA-*b*-NIPAM)-S-(C=S)-S-C_12_H_25_, APS, TFEMA and EGDMA will undergo polymerization in nanoreactors to generate cross-linked nanoparticles. Figure 4 shows the ^19^F-NMR spectrum of lyophilized triblock copolymer P(DMA_33_-*b*-NIPAM_163_-*b*-TFEMA_50_)-S-(C=S)-S-C_12_H_25_. The characteristic signals of the PTFEMA segment (−73.8 ppm –C*F*_3_ in side chain PTFEMA) can be seen. The particle morphology, shown by the transmission electron microscopy (TEM) and scanning electron microscopy (SEM) images in Figure 5 and Figure 6, clearly suggests that stable and monodisperse spherical nanoparticles were formed. The particles’ corona was formed by P(DMA-*b*-NIPAM) and the cross-linked PTFEMA block forms the hydrophobic core.

### 2.3. Influence of the Amount of APS on Aqueous Polymerization

**Figure 7:**
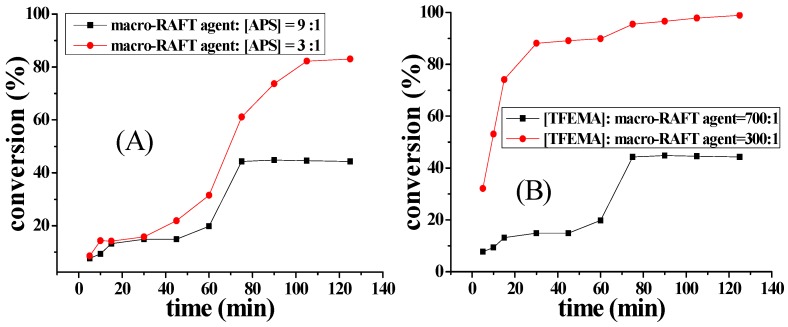

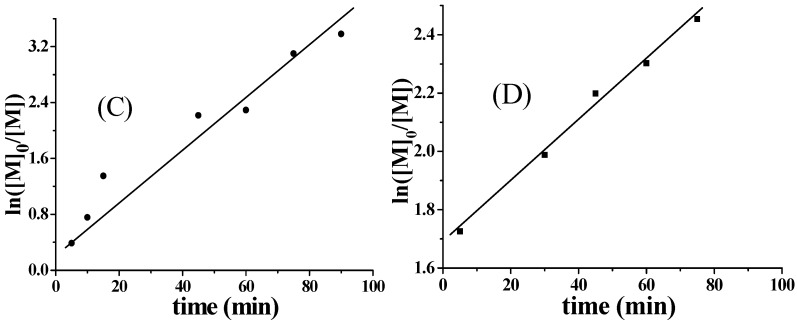
Evolutions of TFEMA conversion versus time for RAFT-mediated aqueous polymerization. Polymerization conditions: (**A**) macro-RAFT agent:[TFEMA] = 1:700, [EGDMA]:[TFEMA] = 5:100, 70 °C; (**B**) macro-RAFT agent:[APS] = 3:1, [EGDMA]:[TFEMA] = 5:100, 70 °C; (**C**) macro-RAFT agent:[TFEMA] = 1:300, macro-RAFT agent:[APS] = 3:1 [EGDMA]:[TFEMA] = 5:100, 70 °C; (**D**) macro-RAFT agent:[TFEMA] = 1:300, macro-RAFT agent:[APS] = 9:1, [EGDMA]:[TFEMA] = 5:100, 70 °C.

Several sets of experiments using different amounts of APS were carried out. Samples were withdrawn at regular time intervals for determination of monomer conversion and particle size. The particle size and the distribution of highly diluted samples were measured by DLS. Figure 7 shows the evolution of TFEMA conversion versus time for RAFT-mediated aqueous polymerization using P(DMA_77_-*b*-NIPAM_73_)-S-(C=S)-S-C_12_H_25_ as macro-RAFT agent.

As shown in Figure 7A the final monomer conversion was 44.3% when the molar ratio of APS and chain transfer agent was 1:9. The final monomer conversion reached 94.2% upon changing the molar ratio of APS and chain transfer agent from 1:9 to 1:3. The low APS concentration process could not generate enough radicals to initiate the reaction, resulting in low monomer conversion. By increasing the amount of APS, the polymerization rate increased due to the increase of the number of radicals. Figure 7B shows the evolution of TFEMA conversion versus time, keeping the same amount of initiator. In the two experiments, the molar ratio of TFEMA and macro-RAFT agent was varied, but the amount of initiator was held constant. In the later stages of the reaction, there was not enough initiator to initiate the reaction. As a result, the conversion of the reaction with more monomer was low [28].

### 2.4. Influence of Amount of Cross-Linker on Nanoparticles

**Figure 8:**
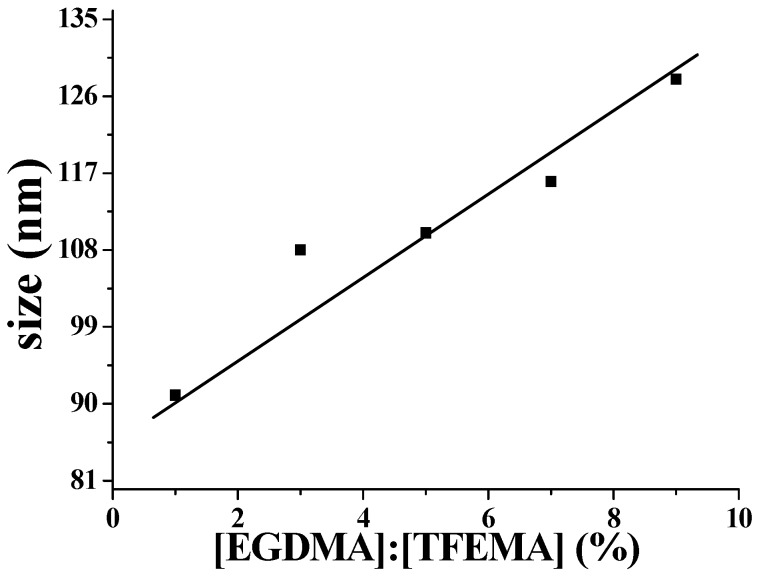
Relationship between particle size and amount of EGDMA added during RAFT-mediated polymerization. Polymerization conditions: macro-RAFT agent:[TFEMA] = 1:700, macro-RAFT agent:[APS] = 3:1, 70 °C, 120 min.

Several experiments were conducted with adding different amounts of cross-linker EGDMA. During this process, P(DMA_46_-*b*-NIPAM_31_)-S-(C=S)-S-C_12_H_25_ was used as macro-RAFT agent. From the results we can conclude that the amount of the EGDMA has a significant influence on particle size. As shown in Figure 8, particle size increased linearly with increasing amount of EGDMA. This is because the increase of amount of cross-linker leads to high cross-linking density. As a result, the number of block copolymers connected on each nanoreactor is also increased, leading to larger particle size.

### 2.5. Influence of Amount of TFEMA on the Nanoparticles

**Figure 9:**
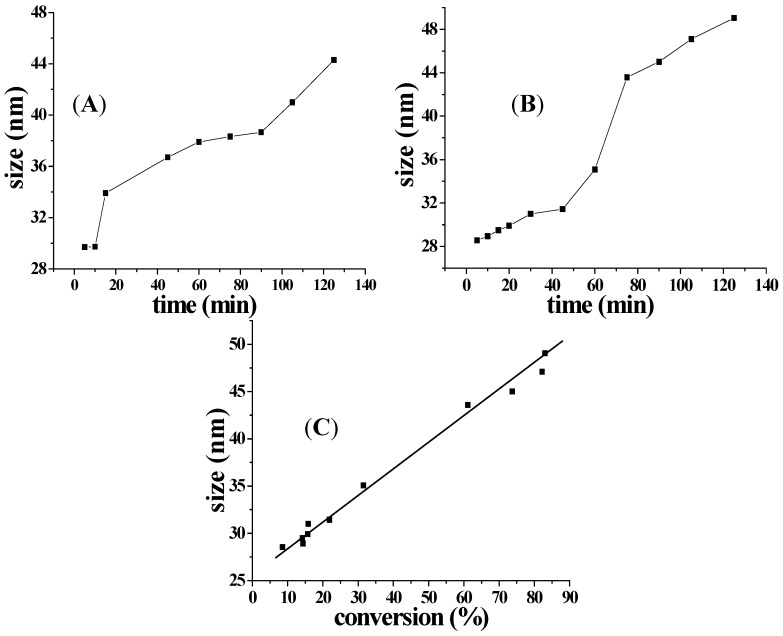
Evolution of particle size measured by DLS at 25 °C versus time or conversion for RAFT-mediated aqueous polymerization. Polymerization conditions: (**A**) macro-RAFT agent:[TFEMA] = 1:700, macro-RAFT agent:[APS] = 9:1, [EGDMA]:[TFEMA] = 5:100, 70 °C; (**B**) macro-RAFT agent:[TFEMA] = 1:700, macro-RAFT agent:[APS] = 3:1, [EGDMA]:[TFEMA] = 5:100, 70 °C; (**C**) macro-RAFT agent:[TFEMA] = 1:700, macro-RAFT agent:[APS] = 3:1, [EGDMA]:[TFEMA] = 5:100, 70 °C.

The effect of amount of TFEMA on particle size was investigated using P(DMA_46_-*b*-NIPAM_31_)-S-(C=S)-S-C_12_H_25_ as macro-RAFT agent. Intensity weighted distributions of the hydrodynamic diameter were obtained by DLS. Figure 9 shows the evolution of particle size versus time or TFEMA conversion. As the reaction proceeded, the particle size in diameter gradually increased from 28.55 to 49.04 nm. This is because the monomer conversion gradually increased as the reaction proceeded, and the core of spherical particle formed by cross-linking PTFEMA will expand with the increase of monomer conversion. Higher monomer conversion means more monomer entered into nanoreactors for polymerization resulting in larger particle size.

### 2.6. Thermo-Responsive Characterization of Cross-Linked Nanoparticles

**Figure 10:**
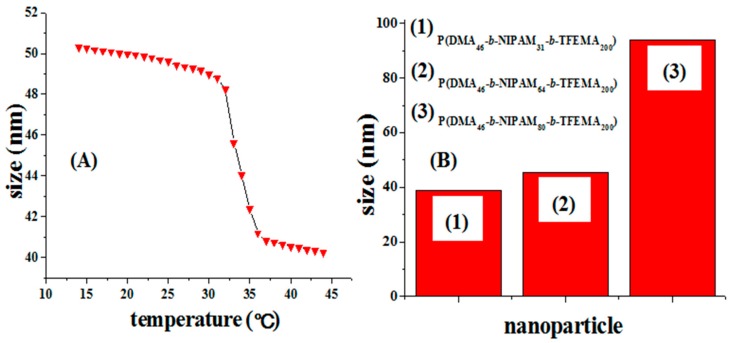
DLS-based size measurement of P(DMA_33_-*b*-NIPAM_163_-*b*-TFEMA_50_)-S-(C=S)-S-C_12_H_25_ (**A**) at 0.5 mg/mL concentration in water at different temperature; (**B**) the particle size of P(DMA-*b*-NIPAM-*b*-TFEMA)-S-(C=S)-S-C_12_H_25_ with different degree of polymerization of PNIPAM at 25 °C.

Figure 10A shows the effect of temperature on the particle size in the range of 14–44 °C. From 14 to 32 °C the particle diameter underwent small changes, but in the 32–37 °C range, the particle size was dramatically reduced from 48 to 41 nm. In the range of 35–44 °C, the particle size remained stable, indicating that the cross-linked nanospheres are temperature sensitive.

Meanwhile, it suggests that the LCST of the nanospheres is 32 °C. PNIPAM segments are in a state of complete dissolution and stretching when the temperature is below 32 °C. As a result, the DLS-based size of the nanospheres is large. When the temperature is raised to the LCST of PNIPAM segments, PNIPAM segments begin to shrink rapidly leading to smaller particle size. After the complete shrinkage of PNIPAM segments at 37 °C, particle size will not change with the increase of temperature. This result proves that PNIPAM is connected with the cross-linked nanospheres, and it can freely stretch or shrink. To further prove this result, we synthesized the nanospheres with the same degree of polymerization of PDMA and PTFEMA and only changed the degree of polymerization of PNIPAM. As shown in Figure 10B, the particle size increased with the increase of degree of polymerization of PNIPAM, indicating that PNIPAM is connected with the cross-linked nanospheres outside.

## 3. Materials and Methods

### 3.1. Materials and Reagents

All reagents and solvents were of analytical grade and used as received unless otherwise stated. Ethyleneglycol dimethacrylate (EGDMA, 98%, Aladdin, Shanghai, China) and *N*,*N*-dimethylacrylamide (DMA, 98%, Aladdin) were passed through a column of basic alumina to remove inhibitor. *N*-isopropylacrylamide (NIPAM, 99%, Sigma-Aldrich, St. Louis, MO, USA) was recrystallised from a 70:30 hexane/toluene mixture prior to use. Azobisisobutyronitrile (AIBN, Aladdin, 98%) was recrystallized twice from methanol prior to use. (2,2,2-Trifluoroethyl) methacrylate (TFEMA) supplied by Wei Hai Newera (Weihai, China) was distilled under reduced pressure prior to use. 2-([(Dodecylsulfanyl)carbonothioyl]sulfanyl)-propanoic acid (DOPAT) was synthesized according to a literature protocol [29].

### 3.2. NMR Analysis

The ^1^H-NMR and ^19^F-NMR analysis was performed on an AVANCE III 400 MHz digital NMR spectrometer (Bruker BioSpin, Karlsruhe, Germany) using deuterated acetone as solvent with tetramethylsilane as the internal standard at room temperature.

### 3.3. GPC Analysis

The molecular weights and dispersity (Ð) were determined by gel permeation chromatography (GPC) at 35 °C with tetrahydrofuran (THF) as the eluent at a flow rate of 1.0 mL/min. Narrow-polydispersity polystyrene was used as calibration standard. The system was equipped with a Model 1525 HPLC pump (Waters, Milford, MA, USA) and a Waters Model 2414 refractive index (RI) detector.

### 3.4. TEM Characterization

Transmission electron microscopy (TEM) observation was performed using a JEOL-1400 electron microscope (Jeol, Tokyo, Japan). A typical TEM grid preparation was as follows: A particle solution was diluted with MilliQ water to approximately 0.10 wt %. A formvar precoated copper TEM grid was covered with a drop of the solution for 60 s, and counterstained with 3% uranyl acetate (5 μL) for 20 s.

### 3.5. SEM Characterization

Morphology of lyophilized particle powder analysis was conducted by scanning electron microscopy (SEM, S-2500, Hitachi Seiki, Tokyo, Japan).

### 3.6. DLS Characterization

A Zetasizer Nano-ZS90 (Malvern Instruments, Malvern, England) was used for dynamic light scattering (DLS) characterization to measure particle size (hydrodynamic diameter Z-Ave) and particle size distribution. The sample refractive index (RI) was set at 1.59 for polystyrene.

### 3.7. Synthesis of Macro-RAFT Agent of PDMA_77_-S-(C=S)-S-C_12_H_25_

In a typical synthesis, a round-bottomed flask was charged with DMA (15.00 g; 1.51 × 10^−1^ mol), DOPAT (0.5289 g; 1.51 × 10^−3^ mol), AIBN (0.0502 g, 3.06 × 10^−4^ mol; CTA/initiator molar ratio = 4.9) and dioxane (23.00 g). The sealed reaction vessel was purged with nitrogen and placed in a pre-heated oil bath at 65 °C for 7 h. The solution was cooled in an ice bath, diluted with dioxane. The polymer was recovered by precipitation in diethyl ether, filtration and drying under vacuum for 48 h at 35 °C. ^1^H-NMR indicated the actual degree of polymerization of 77 for the PDMA macro-CTA. *M*_n_ = 7900 g·mol^−1^ and *M*_w_/*M*_n_ = 1.12, as determined by GPC.

### 3.8. Synthesis of Thermo-Responsive Diblock P(DMA_77_-b-NIPAM_73_)-S-(C=S)-S-C_12_H_25_

In a typical synthesis, a round-bottomed flask was charged with NIPAM (1.1338 g, 1.00 × 10^−2^ mol), PDMA_77_-S-(C=S)-S-C_12_H_25_ (1.00 g; 1.25 × 10^−4^ mol), AIBN (0.0025 g, 1.52 × 10^−5^ mol; CTA/initiator molar ratio = 8.2) and dioxane (3.50 g). The sealed reaction vessel was purged with nitrogen and placed in a pre-heated oil bath at 65 °C for 24 h. The solution was cooled in an ice bath, diluted with dioxane. The polymer was recovered by precipitation in a mixture hexane:toluene (90:10), filtration and drying under vacuum for 48 h at 35 °C. ^1^H-NMR indicated the NIPAM actual degree of polymerization of 73 for the P(DMA_77_-*b*-NIPAM_73_)-S-(C=S)-S-C_12_H_25_. *M*_n_ =14900 g·mol^−1^ and *M*_w_/*M*_n_ = 1.15, as determined by GPC.

### 3.9. Aqueous RAFT-Mediated Polymerization of Cross-Linked TFEMA Nanoparticles in the Presence of P(DMA-b-NIPAM)-S-(C=S)-S-C_12_H_25_

The following aqueous polymerization protocol conducted at 15% *w*/*w* solids and targeting PDMA_77_-*b*-PNIPMA_73_-*b*-PTFEMA_700_ is typical. Water (9.59 g) and P(DMA_77_-*b*-NIPAM_73_)-S-(C=S)-S-C_12_H_25_ (0.19 g; 1.17 × 10^−5^ mol) were added to a round-bottomed flask and stirred in an ice bath for 1 h to allow the complete solubilization of the diblocks. TFEMA (1.4179 g; 8.44 × 10^−3^ mol), EGDMA (0.0833 g; 4.21 × 10^−4^ mol), APS (0.0009 g; 3.95 × 10^−6^ mol) were added to the flask. The solution was deoxygenated with nitrogen and immersed in a water bath at 35 °C for 10 min to allow the formation of micelles. Then the solution was heated at 70 °C for 120 min.

## 4. Conclusions

In summary, thermo-responsive and cross-linked PTFEMA nanospheres were successfully prepared. Firstly, RAFT solution polymerization was used to synthesize a thermo-responsive diblock copolymer P(DMA-*b*-NIPAM)-S-(C=S)-S-C_12_H_25_ with low dispersity and well-controlled molecular weight. The ^1^H-NMR spectral analysis confirmed that copolymers were successfully synthesized. Gel permeation chromatography was conducted to demonstrate the narrow molecular weight dispersity. Then we gave evidence that the thermo-responsive diblock copolymer can turn into nanoreactors when the temperature reaches 32 °C. We proceeded a successful RAFT-mediated aqueous polymerization of TFEMA using P(DMA-*b*-NIPAM)-S-(C=S)-S-C_12_H_25_ trithiocarbonate as both a stabilizer and a macro-RAFT agent. During the process of aqueous polymerization, the monomer conversion increased with increasing the molar ratio of initiator to chain transfer agent. The particle size increased with increasing the monomer conversion. The particle size of the cross-linked nanospheres quickly decreased when the temperature reached 32 °C showing temperature sensitivity.

## Figures and Tables

**Table 1:** Data for the reversible addition-fragmentation chain transfer (RAFT) mediated polymerization of *N*,*N*-dimethylacrylamide (DMA) at 65 °C in dioxane.

[DMA]:[DOPAT]	M_n,target_^a^ (g/mol)	x ^b^ (%)	M_n,theory_^c^ (g/mol)	M_n,GPC_^d^	Ð ^e^
35:1	3800	97	3700	3900	1.09
100:1	10,250	84	8700	7500	1.11
150:1	15,200	73	11,200	10,700	1.09

^a^ target molecular weight (M_n_) of PDMA; ^b^ conversion of DMA calculated by gravimetry; ^c^ calculated from the equation: $M_{n,\mathrm{theory}}=\frac{\left[\mathrm{DMA}\right]}{\left[\mathrm{DOPAT}\right]}\times M_{\mathrm{DMA}}\times \mathrm{conversion}+M_{\mathrm{DOPAT}}$ where M_DOPAT_ is the molecular weight of 2-([(dodecylsulfanyl)carbonothioyl]sulfanyl)-propanoic acid (DOPAT); ^d^ THF was used as eluent at a flow rate of 1.0 mL/min; ^e^ dispersity of PDMA.

**Table 2:** Data for the RAFT-mediated polymerization of *N*-isopropylacrylamide (NIPAM) at 65 °C in dioxane.

[NIPAM]:[macro-CTA]	M_n,target_^a^ (g/mol)	x ^b^ (%)	M_n,theory_^c^ (g/mol)	M_n,GPC_^d^	Ð ^e^
40:1	8100	92	7700	7200	1.07
100:1	17,200	78	12,400	14,800	1.16
250:1	31,800	64	20,300	22,000	1.17

^a^ target molecular weight (M_n_) of PDMA-*b*-PNIPAM; ^b^ conversion of NIPAM calculated by gravimetry; ^c^ calculated from the equation: $M_{n,\mathrm{theory}}=\frac{\left[\mathrm{NIPAM}\right]}{\left[\mathrm{macro}-\mathrm{CTA}\right]}\times M_{\mathrm{NIPAM}}\times \mathrm{conversion}+M_{n,\mathrm{PDMA}}$ where M_n,PDMA_ is the molecular weight of the PDMA_33_ macro-CTA; ^d^ THF was used as eluent at a flow rate of 1.0 mL/min; ^e^ dispersity of PDMA-*b*-PNIPAM.
